# Spatial and Temporal Dynamics of Fucoid Populations (*Ascophyllum nodosum* and *Fucus serratus*): A Comparison between Central and Range Edge Populations

**DOI:** 10.1371/journal.pone.0092177

**Published:** 2014-03-20

**Authors:** Rita M. Araújo, Ester A. Serrão, Isabel Sousa-Pinto, Per Åberg

**Affiliations:** 1 Interdisciplinary Centre of Marine and Environmental Research (CIIMAR/CIMAR), University of Porto, Porto, Portugal; 2 Centre of Marine Sciences, University of Algarve (CIMAR-Algarve), Campus of Gambelas, Faro, Portugal; 3 Department of Biology, Faculty of Sciences, University of Porto, Porto, Portugal; 4 Department of Biological and Environmental Sciences, University of Gothenburg, Göteborg, Sweden; Bangor University, United Kingdom

## Abstract

Persistence of populations at range edges relies on local population dynamics and fitness, in the case of geographically isolated populations of species with low dispersal potential. Focusing on spatial variations in demography helps to predict the long-term capability for persistence of populations across the geographical range of species’ distribution. The demography of two ecological and phylogenetically close macroalgal species with different life history characteristics was investigated by using stochastic, stage-based matrix models. Populations of *Ascophyllum nodosum* and *Fucus serratus* were sampled for up to 4 years at central locations in France and at their southern range limits in Portugal. The stochastic population growth rate (λ*_s_*) of *A. nodosum* was lower and more variable in central than in southern sites whilst for *F. serratus* this trend was reversed with λ*_s_* much lower and more variable in southern than in central populations. Individuals were larger in central than in southern populations for both species, which was reflected in the lower transition probabilities of individuals to larger size classes and higher probability of shrinkage in the southern populations. In both central and southern populations elasticity analysis (proportional sensitivity) of population growth rate showed that fertility elements had a small contribution to λ*_s_* that was more sensitive to changes in matrix transitions corresponding to survival. The highest elasticities were found for loop transitions in *A. nodosum* and for growth to larger size classes in *F. serratus*. Sensitivity analysis showed high selective pressure on individual growth for both species at both locations. The results of this study highlight the deterministic role of species-specific life-history traits in population demography across the geographical range of species. Additionally, this study demonstrates that individuals’ life-transitions differ in vulnerability to environmental variability and shows the importance of vegetative compared to reproductive stages for the long-term persistence of populations.

## Introduction

Understanding the factors that set species geographical borders is a central issue in ecology and evolutionary biology [1] that has received increased attention in the face of the rapid distributional shifts associated with climate change. All species have limits to their geographical ranges beyond which they are not found, presumably because at these locations their tolerances and capacities are constrained or dispersal from source populations is limited [2], but these assumptions have rarely been tested [3]. The geographical ranges of species distributions may result from the interplay between many factors including habitat availability, local environmental conditions, metapopulation dynamics, biological interactions and species-specific traits, such as physiological tolerances, dispersal potential and vital rates [4], [5].

Variations in population structure and dynamics across the species distributional ranges must reflect geographical variation in the dynamics of vital rates [6], [7]. Therefore, to understand the determinants of species ranges it is fundamental to compare the vital rates at range boundaries to those of central populations and to understand how spatial variation in fitness translates into population-level differences in abundance [3]. Such comparisons are surprisingly rare, and even less frequent for southern latitude margins that are more susceptible to change as a consequence of climatic change. Exceptions include empirical comparisons between demographic traits at marginal and core locations for plants [7], [8], and marine organisms [9], [10]. Studies on seaweeds, compared isolated [11] and integrated [12] demographic traits at central and southern range locations, or developed demographic models for central and marginal populations. However these studies did not include the most marginal populations [13]. Understanding the basis of the contrasting dynamics of edge and central populations will increase the reliability of models on the current, likely threatened e.g. [14], [15], status of populations at range margins and to predict their long-term response to current and future environmental conditions.

Geographically isolated populations of dioecious species with short direct dispersal are more likely to be affected by disturbance, especially if they are at the margins of their distributional range. Among this group of species are the two canopy forming fucoid seaweeds *Ascophyllum nodosum* and *Fucus serratus* that occur on sheltered intertidal shores in the north Atlantic. These are two ecologically and phylogenetically close foundation species with very different life-spans and phylogeographical histories. *A. nodosum* is a long-lived species able to produce aggregated clonal individuals by vegetative sprouting from its holdfast (base), with slow growth rate and an estimated maximum life span of a genetical individual of hundreds of years [16], whereas *F. serratus* does not produce clonal copies from the holdfast and has a shorter life span of about 3 to 4 years [17]. Patterns of genetic diversity at the southern range edge are also very different for both species [18], [19], [20]. Lower genetic diversity and high between population differentiation have been reported for *F. serratus* populations of the Iberian Peninsula when compared with central ones [18], [19] which might limit the evolutionary potential of such marginal populations [21]. Similar genetic diversity and very low differentiation were found across the entire range of distribution of *A. nodosum* in the Atlantic, with exception of Brittany where higher values were reported [20]. This shows that genetic diversity is not reduced nor distinct at the southern edge. Population structure and demographic variables of both species at marginal and central locations are also different. *A. nodosum* marginal populations exhibited differentiated phenotypic traits in relation to central populations with higher values of reproductive output and densities and lower individuals’ sizes, while *F. serratus* core and border populations did not show differences in vital rates [12].

The aim of the present study is to compare the demography of marginal and central populations of *A. nodosum* and *F. serratus* using stochastic matrix population models. These models are powerful tools to investigate the demography of stage-structured populations and allow the incorporation of temporal and spatial sources of variability in population dynamics [22]. Environmental stochasticity affects population dynamics and evolution of life-history traits and thus stochastic matrix models that include variations in vital rates due to temporal random fluctuations in the environment, were used in this study. Stochastic stage-based demographic models for the two species are developed and analyzed with focus on stochastic population growth rate, mean stable stage distribution, as well as specific sensitivity and elasticity of the stochastic population growth rate to changes in stage-specific vital rates. More specifically, we hypothesize that the stochastic population growth rate should be lower and more variable in the marginal than in the central populations and that the sensitivity of the stochastic population growth rate should be more sensitive to changes in fertility in the marginal populations. Furthermore, these analyses aim to contribute to global understanding of demographic contrasts in marginal versus central populations and their consequences for persistence of populations at their low-latitude distributional limits.

## Materials and Methods

### Study Site

This study was conducted on the northern coast of Portugal and the northern coast of Brittany in France covering, respectively, the southern-edge and central populations of *F. serratus* and *A. nodosum*. These species have maintained stable isolated southern-edge populations on the northern coast of Portugal for at least the last 50 years [23]. In Portugal, the study of *F. serratus* was performed in Viana do Castelo (41°41′27″N; 8°50′57″W) and Amorosa (41°38′26″N; 8°49′22″W) and for *A. nodosum* only in Viana do Castelo, the southernmost population in the eastern Atlantic (41°41′49″N; 8°51′08″W). In the French coast, two populations of both species were sampled, in Roscoff (48°43′38″N; 3°59′22″W) and in Santec (48°42′46″N; 4°01′23″W).

The central populations of both species were located in sheltered bays, protected by groups of coastal islands while the southern edge populations are on an open coast, exposed to prevailing NW swell. However, the area of the shore where *A. nodosum* and *F. serratus* occur is protected from the direct action of waves by a large extension of rocks located in front of their areas. This is demonstrated by the prevalence, in these sheltered zones of the shore, of brown algae assemblages dominated by *Pelvetia canaliculata*, *F. spiralis* and *F. vesiculosus* and *A. nodosum*.

No specific permissions were required to perform this study since it did not involve collection of specimens and none of the species studied had endangered or protected status.

All sites were strongly dominated by fucoid canopy at mid-intertidal sheltered shores. *A. nodosum* was found in the mid-high intertidal zone and *F. serratus* in the mid-low levels. The maximum tidal amplitude is approximately 4.0 m on the northern Portuguese coast and 9.0 m in the northern part of Brittany. Mean annual temperatures recorded during the study period under the *A. nodosum* canopy were 16°C in Portugal and 11°C in France [12].

### Sampling Procedure

For each location (Portugal and France), at each site (Viana do Castelo and Amorosa in Portugal, and Roscoff and Santec in France) and for each species, three 1×1 m quadrats were randomly selected and marked in the corners with plastic tags and screws. However, for *A. nodosum* in Portugal only Viana do Castelo was sampled since it is the single southern border population that exists. The nearest known population occurs 10 s of kms further north, in Spain. For this reason, twelve 1×1 m plots were randomly spread along the 2000×30 m total distribution of the population in Viana do Castelo. Inside each quadrat and at each sampling occasion all the individuals were mapped. Individuals with a size large enough to hold a plastic cable tie or a glass bead tag were tagged. The basic information needed for the size structured matrix model is the fate of individuals, that is, if they survive or not. If they survive, then the change in size is important in order to calculate the probability of transition of individuals from one size class to another. Further, the number of recruits is needed to estimate the fertility. For that reason all new recruits within each quadrat were registered at each sampling occasion. To estimate individual size, the maximum length (l) and circumference (c) (in cm) of each individual were measured. The predictive equations previously obtained for each species at each location [12], for the relationship between algal volume [length of the longest frond × maximum circumference squared (lc^2^)] and dry weight, were used to calculate the dry biomass (in g) of all the individuals measured in the field. Sampling was done from 2005 to 2009 at variable dates, depending on the knowledge available about each species and each location prior to the start of the experiment. For *A. nodosum* in France, based on previous personal observations about the occurrence of a single reproductive period yearly, sampling was done once a year from 2006 to 2009 (summer). Because similar information was not available for *F. serratus* in France, sampling was done twice a year during the first year (2006–2007) (winter and summer) showing that the reproductive period was restricted to few months per year. Thus, sampling was done yearly in the following sampling occasions (summer). For Portugal, no information about the reproductive period of either species was available. *A. nodosum*’s receptacles were present all year around and thus sampling was performed 3 times per year in 2005 (winter, spring and summer). In 2006 sampling was done twice a year (winter and summer). Additional in-situ observations showed that gamete release occurred mainly between October and February, thus between 2007 and 2009 sampling was done once a year (summer). For *F. serratus* in Portugal, sampling was done twice a year between 2006 and 2008 (winter and summer) and once a year in 2009, since mature receptacles were mainly observed between May and September (end of summer).

**Figure 1:**
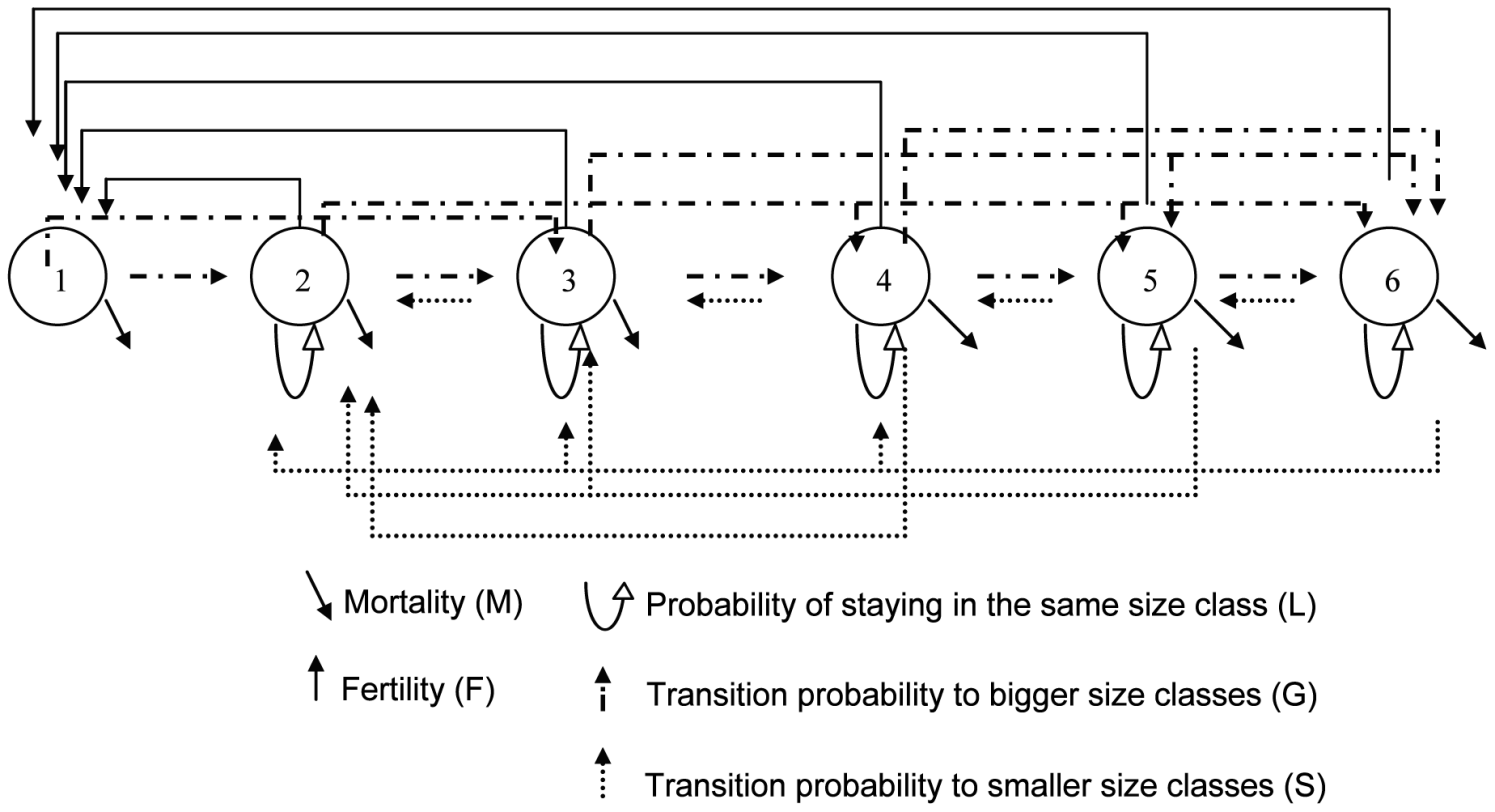
Life cycle graph for *A. nodosum* and *F. serratus* showing the different stage classes and all possible transitions. Individuals in a size class (2–6) that survive can grow (G), shrink (S) or stay in the same size class (L). Class 1 includes new recruits that, if survive, enter class 2. Individuals in class 3–6 are reproductive.

For the demographic analyses, individuals were divided into six classes: the first class included only the new recruits; individuals in this class can only grow to a larger class or die. The other classes were based on individual size (<5 g; 5–15 g; 15–54 g; 54–190 g; >190 g), following [24] and where individuals larger than 5 g were assumed to be fertile. It was not possible to associate recruits to a given fertile alga in the field. For that reason an indirect method was used to calculate the fertility values for a given size class. The individual fertility values were calculated as contributions to the observed number of recruits, for algae in size-classes 3–6 in proportion to their biomass. The assumption that fertility is proportional to adult biomass is supported by results in earlier papers [13], [25], [26], [27]. Projection matrices were constructed based on the life cycle graph shown in Fig. 1.

### Model

#### The matrix model

Matrix population models are structured models where the population is divided into discrete stages, e.g. age, size or natural life-cycle stages or a combination of these types of stages. The population matrix describes the transition probability from one stage to another and the probability to stay in the same stage as well as the stage specific fertility. The change in population size can be projected in discrete time steps and there are numerous analyses that can be applied to the model (see [22] for a review). A stochastic matrix model is usually based on a finite set of projection matrices {**A**_0_, **A**_1_, …, **A**_k_}, each representing a specific environment (e.g. here year and country). The stochastic process can be described by a first-order Markov chain of length *T* (*t* = 0, 1, …, *T*), where *T* is a large number (usually thousands of time steps). To model the population growth a matrix **A**_t_ is drawn from the subset of matrices at each time *t*, with a set probability and multiplied by a column vector **n**(*t*) specifying the number of individuals in each stage class at time t. The basic stochastic matrix population model can be expressed as



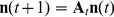
(1)where **n**(*t*) is a population vector and **A**_t_ is the population projection matrix generated by the stochastic environmental process at time step *t*. If the probability distribution of the different environments does not change through time the environment is considered homogenous. The stochastic population growth rate can then be calculated as



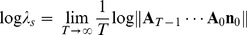
(2)


To our knowledge there are no statistical methods to compare the population growth rate and thus is not possible to calculate the uncertainty in the estimation of this parameter.

#### Model analyses and simulations

Due to the unbalanced temporal sampling there are three matrices describing the first year and two for the second year for *A. nodosum* in Portugal. In year three and four there is only one matrix per year similarly to *A. nodosum* in France where there is only one matrix for each of the three years. For *F. serratus* in Portugal there are two matrices per year for the first and second year and one matrix for the third year. For *F. serratus* in France there are two matrices for the first year and one matrix per year for years two and three. For years with more than one matrix a periodic model was applied to calculate a yearly matrix [28]. The stochastic population growth rate was calculated using yearly matrices where all years had the same probability to appear in the stochastic sample path. For each species and site a mean matrix was calculated from the yearly matrices (Table 1) e.g. for a site with three yearly matrices the mean matrix A_m_ = (A_1_+A_2_+A_3_)/3. The stable stage distribution and reproductive values were calculated as the right and left eigenvectors corresponding to the dominant eigenvalue of the mean matrix for each species and country.

**Table 1:** Mean matrices from stochastic simulations of the *A. nodosum* and *F. serratus* populations in France and Portugal.

***Ascophyllumnodosum*** ** Portugal**											
	0	*F_12_*	0.0176	*F_13_*	0.2425	*F_14_*	0.6670	*F_15_*	1.7894	*F_16_*	5.2537
*G_21_*	0.3283	*L_22_*	0.5546	*S_23_*	0.1860	*S_24_*	0.1172	*S_25_*	0.2378	*S_26_*	0.5534
*G_31_*	0.0285	*G_32_*	0.1279	*L_33_*	0.4621	*S_34_*	0.2148	*S_35_*	0.1106	*S_36_*	0.0402
*G_41_*	0.0035	*G_42_*	0.0243	*G_43_*	0.2564	*L_44_*	0.5476	*S_45_*	0.3467	*S_46_*	0.1485
*G_51_*	0	*G_52_*	0.0008	*G_53_*	0.0140	*G_54_*	0.1108	*L_55_*	0.4672	*S_56_*	0.3278
*G_61_*	0	*G_62_*	0	*G_63_*	0.0002	*G_64_*	0.0012	*G_65_*	0.0172	*L_66_*	0.3603
***Ascophyllumnodosum*** ** France**											
	0	*F_12_*	0	*F_13_*	0.0418	*F_14_*	0.1520	*F_15_*	0.4980	*F_16_*	2.2230
*G_21_*	0.2609	*L_22_*	0.3942	*S_23_*	0.1047	*S_24_*	0.0233	*S_25_*	0.0272	*S_26_*	0.0186
*G_31_*	0	*G_32_*	0.1049	*L_33_*	0.3809	*S_34_*	0.0853	*S_35_*	0.0615	*S_36_*	0.0344
*G_41_*	0	*G_42_*	0.0219	*G_43_*	0.3149	*L_44_*	0.4496	*S_45_*	0.1576	*S_46_*	0.0503
*G_51_*	0	*G_52_*	0	*G_53_*	0.0438	*G_54_*	0.2783	*L_55_*	0.4445	*S_56_*	0.2146
*G_61_*	0	*G_62_*	0	*G_63_*	0.0046	*G_64_*	0.0273	*G_65_*	0.1706	*L_66_*	0.4935
***Fucusserratus*** ** Portugal**											
	0	*F_12_*	0.3104	*F_13_*	2.6177	*F_14_*	5.2028	*F_15_*	13.3929	*F_16_*	19.4457
*G_21_*	0.1458	*L_22_*	0.0825	*S_23_*	0.2734	*S_24_*	0.6538	*S_25_*	1.6188	*S_26_*	4.4581
*G_31_*	0.0063	*G_32_*	0.0312	*L_33_*	0.0456	*S_34_*	0.0411	*S_35_*	0.0400	*S_36_*	0.0102
*G_41_*	0.0022	*G_42_*	0.0368	*G_43_*	0.0849	*L_44_*	0.1273	*S_45_*	0.1064	*S_46_*	0.0749
*G_51_*	0.0008	*G_52_*	0.0220	*G_53_*	0.0804	*G_54_*	0.1615	*L_55_*	0.1295	*S_56_*	0.1266
*G_61_*	0.0004	*G_62_*	0.0015	*G_63_*	0.0111	*G_64_*	0.0367	*G_65_*	0.0955	*L_66_*	0.1136
***Fucusserratus*** ** France**											
	0	*F_12_*	0.0306	*F_13_*	0.656	*F_14_*	1.886	*F_15_*	5.4206	*F_16_*	18.1057
*G_21_*	0.2305	*L_22_*	0.1496	*S_23_*	0.1692	*S_24_*	0.3163	*S_25_*	1.0242	*S_26_*	3.3693
*G_31_*	0.0608	*G_32_*	0.0905	*L_33_*	0.1667	*S_34_*	0.0729	*S_35_*	0.0457	*S_36_*	0.0092
*G_41_*	0.0278	*G_42_*	0.0614	*G_43_*	0.1825	*L_44_*	0.2797	*S_45_*	0.1488	*S_46_*	0.123
*G_51_*	0.0024	*G_52_*	0.0179	*G_53_*	0.1161	*G_54_*	0.2144	*L_55_*	0.2623	*S_56_*	0.305
*G_61_*	0	*G_62_*	0.0016	*G_63_*	0.0022	*G_64_*	0.0429	*G_65_*	0.1318	*L_66_*	0.2078

The transition codes are represented in all matrices to make easier the interpretation of the table. F: Fertility; G: Growth to larger size classes; S: Shrinkage; L: Loop.

#### Sensitivity and elasticity

The sensitivity and elasticity (proportional sensitivity) of the stochastic population growth rate to changes in the matrix elements were calculated using model simulation. Due to the unbalanced temporal sampling the methods in [29] and [30] could not be used directly but the simulations are equivalent to these methods. To calculate the sensitivity of the stochastic population growth rate a small figure (0.0001) was added to one element at the time in all the basic matrices. For years with more than one matrix, the yearly matrix was then calculated with the periodic model and added to the set of yearly matrices. A new stochastic population growth rate was calculated for the perturbed set of matrices using the same stochastic sample path as when calculating the original growth rate. This new stochastic population growth rate minus the original one divided by the size of the perturbation -1 gave the sensitivity for the matrix element that was perturbed. The matrices were then set back to their original values and the next element was perturbed until values for all elements were obtained. The size of the perturbation was decreased until the sensitivity values converged to three significant digits. The elasticity of the population growth rate was calculated in a similar way but with a factor close to unity (1.0001) instead of an absolute perturbation. All calculations and simulations were performed in MATLAB R2011a.

## Results

### Stochastic Population Growth Rate and Stable Stage Distribution

**Figure 2:**
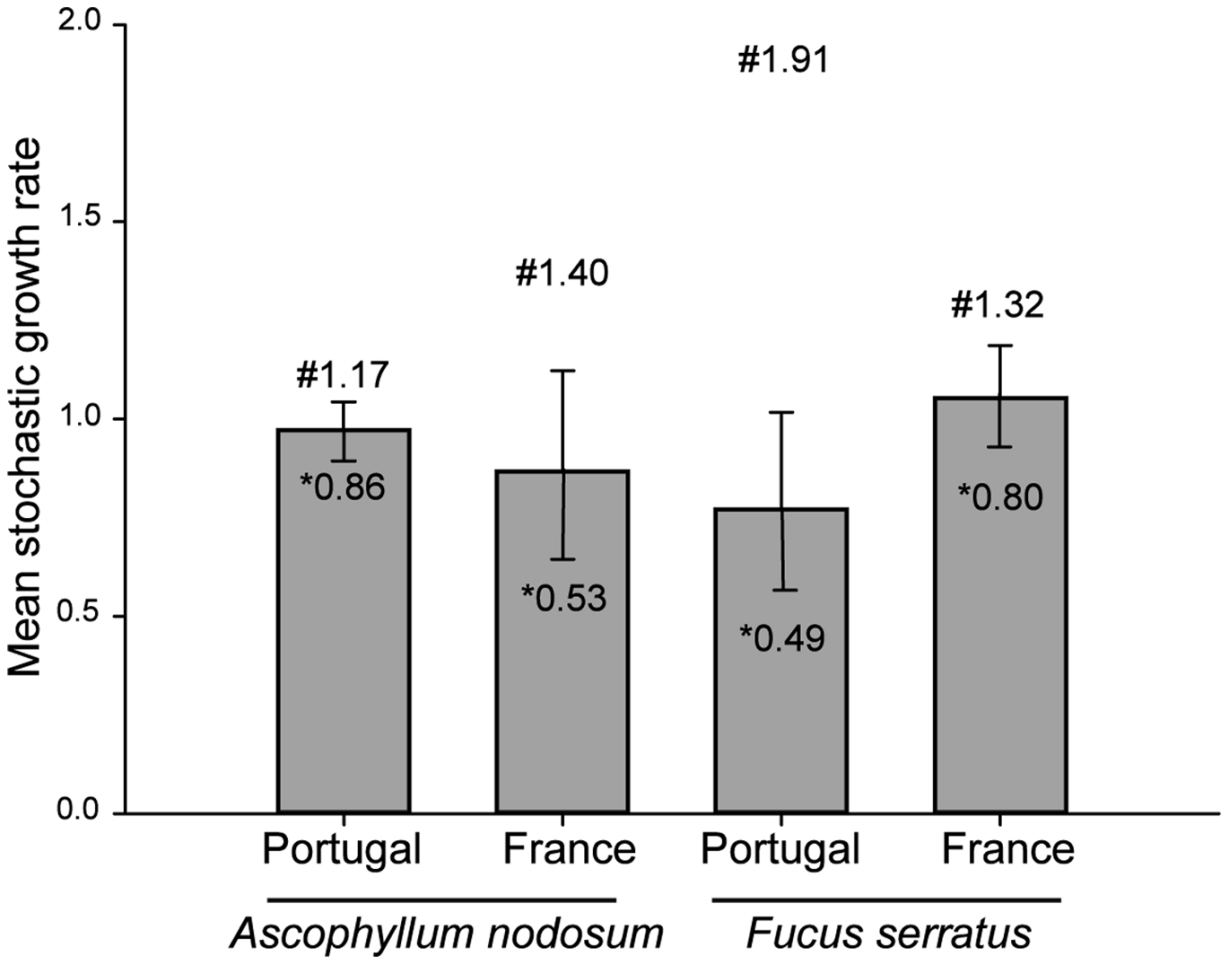
Mean (±SD), minimum (*) and maximum (#) stochastic growth rates (λ_s_) for southern (Portugal) and central (France) *A. nodosum* and *F. serratus* populations.

Model simulations showed that the stochastic population growth rate (λ*_s_*) varied between species and locations. When λ*_s_* is >1 the average population size is growing and when it is <1 the average population size is decreasing. λ*_s_* was higher for *A. nodosum* in Portugal (0.97) than in France (0.87) but lower for *F. serratus* in Portugal (0.77) than in France (1.05) (Fig. 2). All the λ*_s_* were slightly below 1, except for *F. serratus* in France (Fig. 2). For *A. nodosum*, variation in population growth rate was larger in France than in Portugal but the opposite trend was recorded for *F. serratus* (Fig. 2).

**Figure 3:**
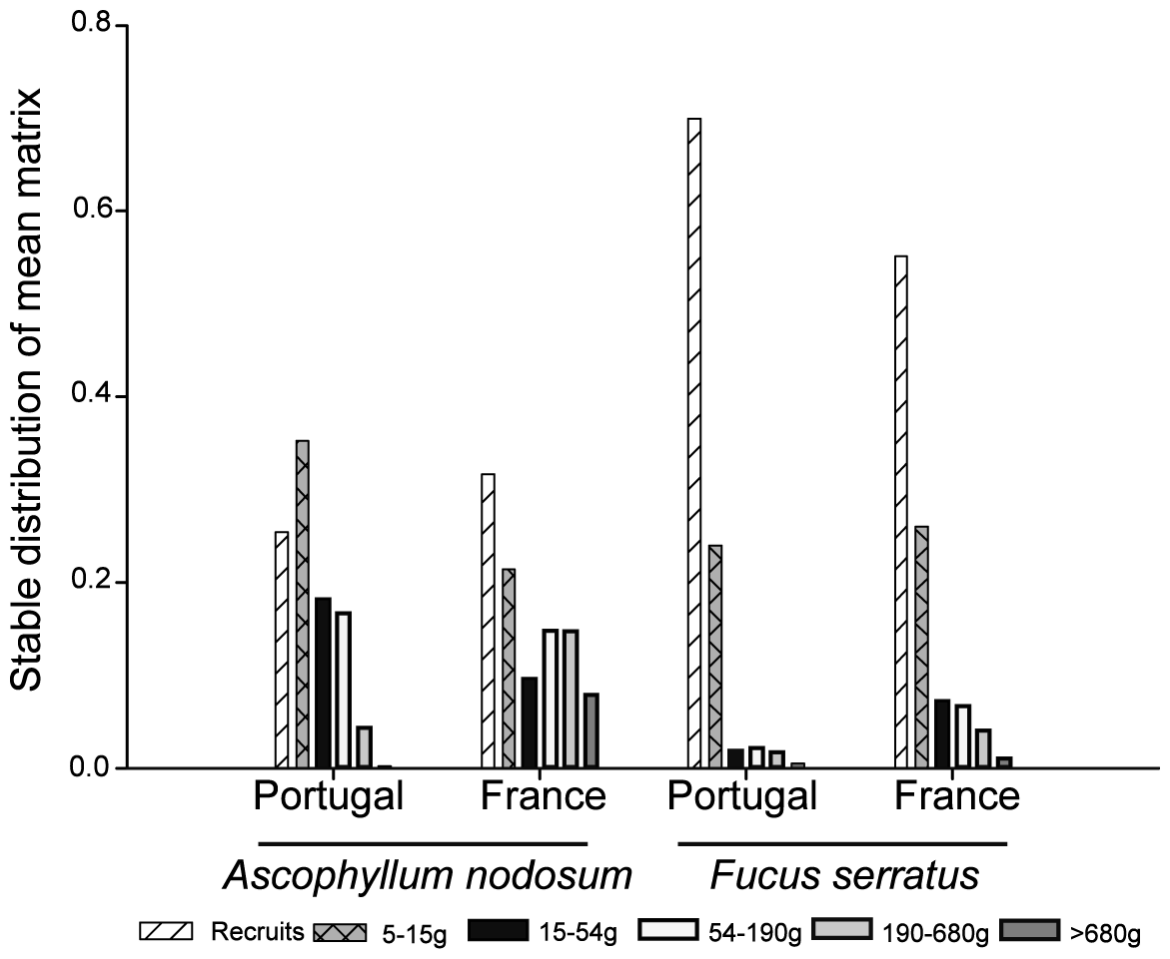
Stable distribution of the mean matrix for southern (Portugal) and central (France) *A. nodosum* and *F. serratus* populations.

The stable stage distribution calculated from the mean matrix showed for both *A. nodosum* and *F. serratus*, that populations in France had higher proportion of individuals belonging to the largest size classes (classes 5 and 6 for *A. nodosum* and classes 3 to 6 for *F. serratus*) than in Portugal. However, all the populations, especially *F. serratus*, both in Portugal and France, were dominated by smaller individuals belonging to classes 1 and 2 (Fig. 3).

### Reproductive Values

**Figure 4:**
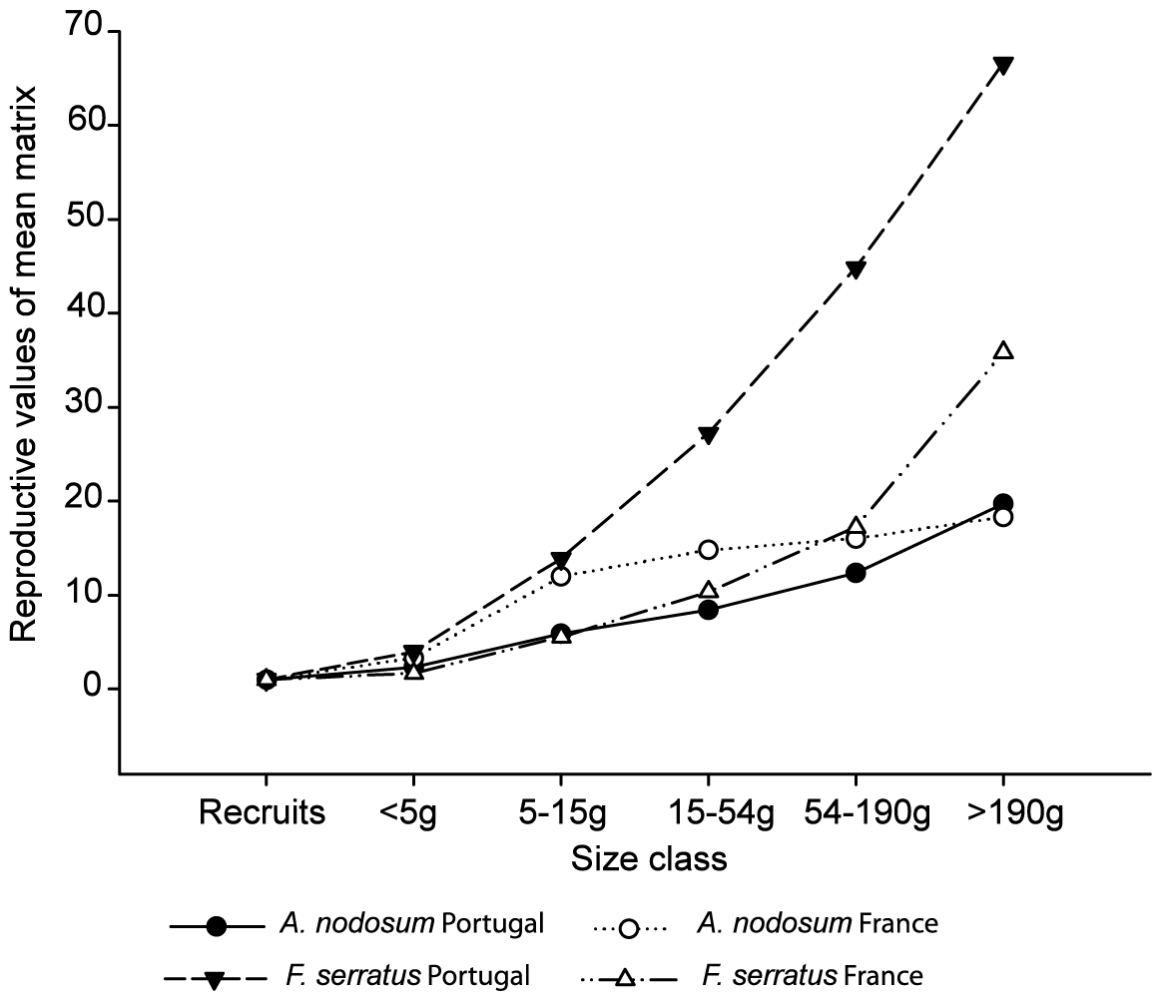
Reproductive values for each stage class of southern (Portugal) and central (France) *A. nodosum* and *F. serratus* populations.

Reproductive values increased with plant size for both species at both locations but more pronouncedly for *F. serratus*, especially in Portugal (Fig. 4). These results were related to the fertilities of each class and the transition probabilities between size classes. Higher fertilities were found in Portugal for both species at all size classes. However, for *A. nodosum* in Portugal the probability of transition to the class with higher fertility (class 6) is very low and the shrinkage to smaller size classes much higher than in France (Table 1). On the contrary, for *F. serratus* the differences in the transition to higher size classes and in the shrinkage of individuals to smaller sizes between locations are not so pronounced, and do not balance the much higher values of fertility found in Portugal (Table 1).

### Elasticity and Sensitivity

**Figure 5:**
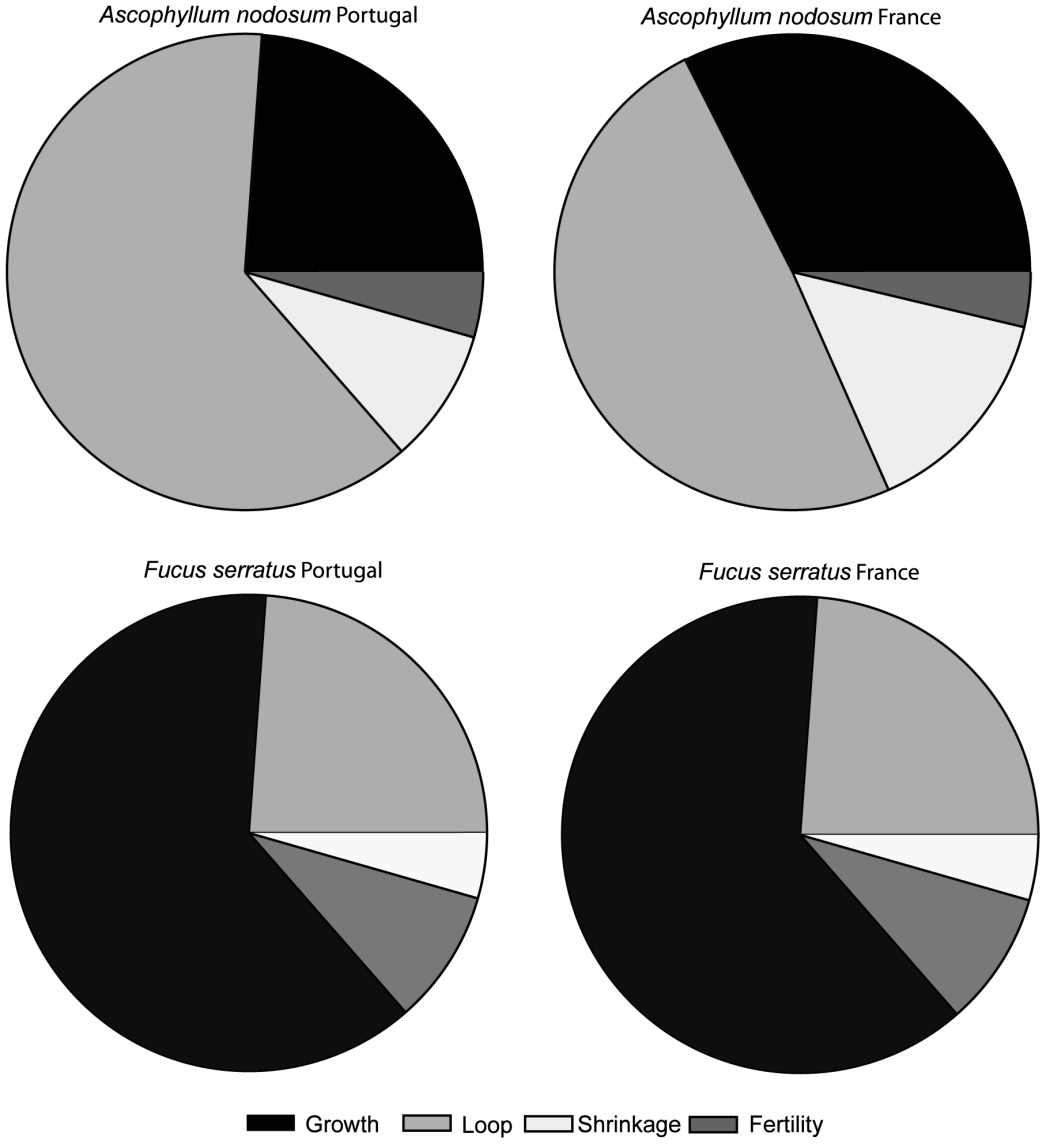
Elasticity of the population growth rate to changes in matrix elements summed over different regions (growth, loop, shrinkage and fertility) for southern (Portugal) and central (France) *A. nodosum* and *F. serratus* populations.

**Figure 6:**
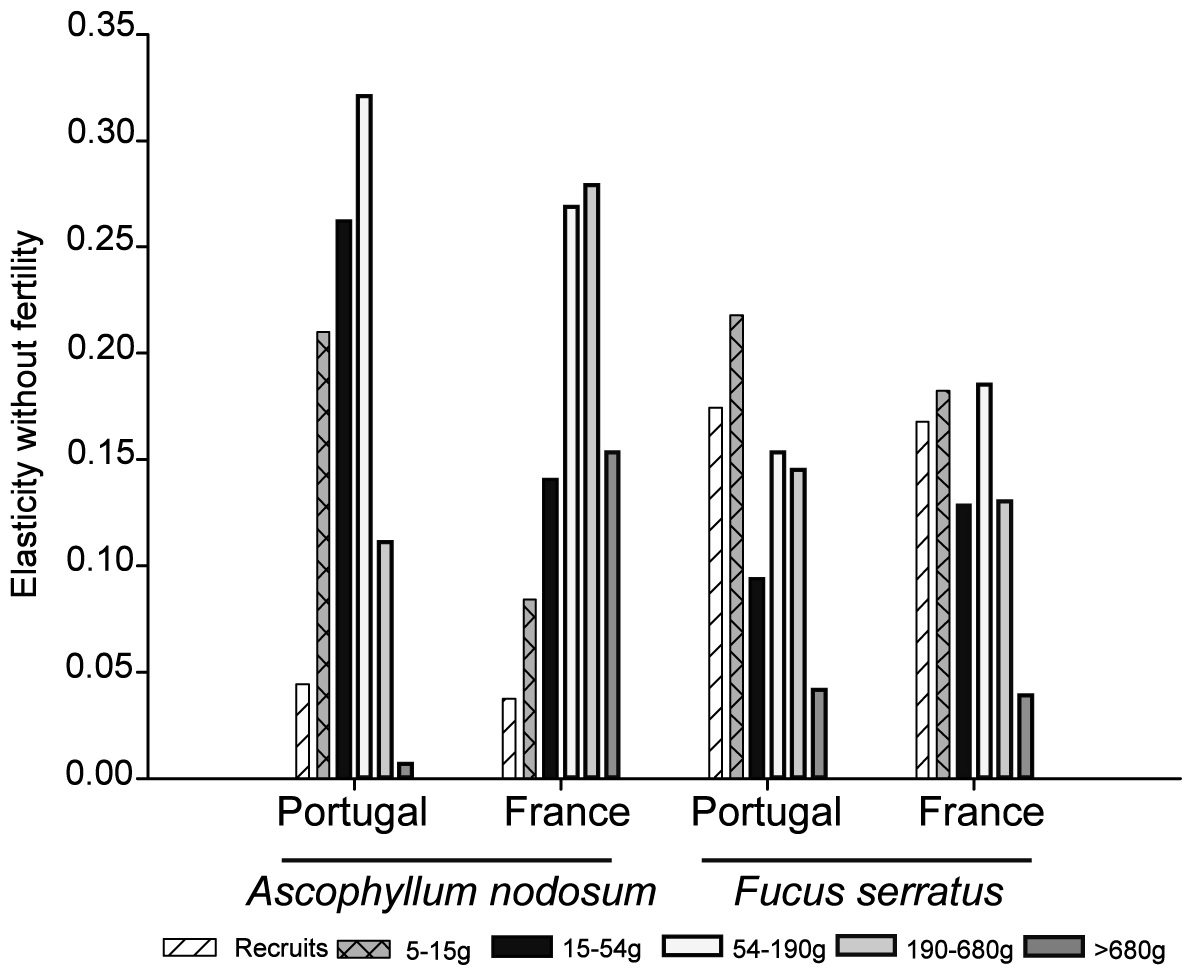
Elasticity of the population growth rate to changes in the survival transitions for each stage class in southern (Portugal) and central (France) *A. nodosum* and *F. serratus* populations.

The pattern of elasticities of the stochastic population growth rate to changes in the matrix elements showed that the loop transition was the most important for the λ*_s_* of *A. nodosum* while the growth transition was the most important for the λ*_s_* of *F. serratus* (Fig. 5). Elasticities were very similar for *F. serratus* populations in France and in Portugal, with the shrinkage transition as the least important for λ*_s_* (Fig. 5). Fertility transitions were very low for *A. nodosum* when compared to *F. serratus* but were slightly higher in Portugal than in France (Fig. 5). For *A. nodosum*, loop transitions were more important in Portugal than in France while growth and shrinkage transitions showed the opposite geographical pattern (Fig. 5). The comparison between the elasticity across size classes, without taking into account the fertility, showed that for *A. nodosum* in France there was an important contribution of the 3 largest size classes (4–6) to λ*_s_* while in Portugal it was classes 2 to 4 that influence λ*_s_* the most (Fig. 6). For *F. serratus* the contribution of each size class to λ*_s_* was more evenly distributed among size classes 1 to 5 for both locations (Fig. 6).

**Figure 7:**
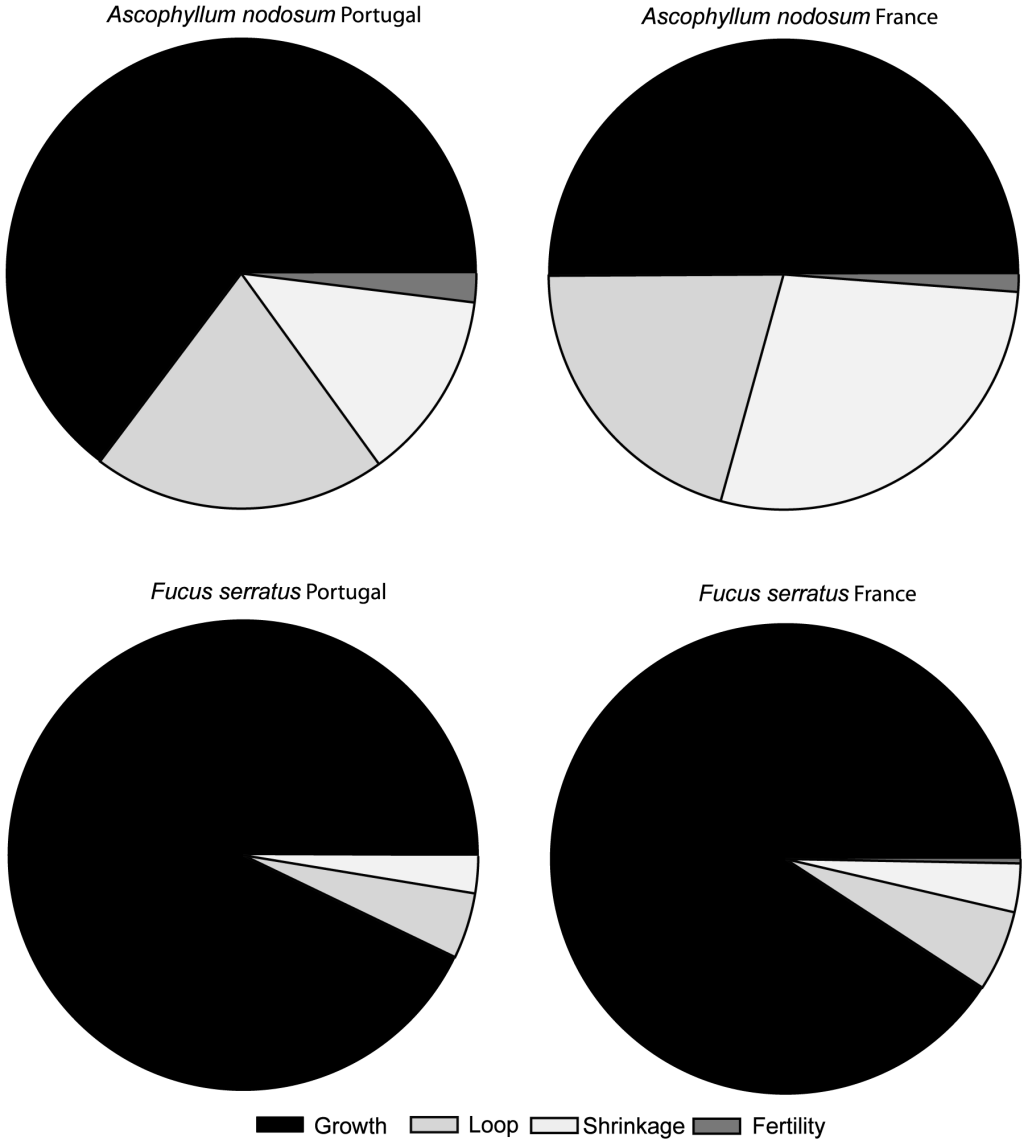
Sensitivity of the population growth rate to changes in matrix elements expressed as relative contributions and summed over different regions (growth, loop, shrinkage and fertility) in southern (Portugal) and central (France) *A. nodosum* and *F. serratus* populations.

**Figure 8:**
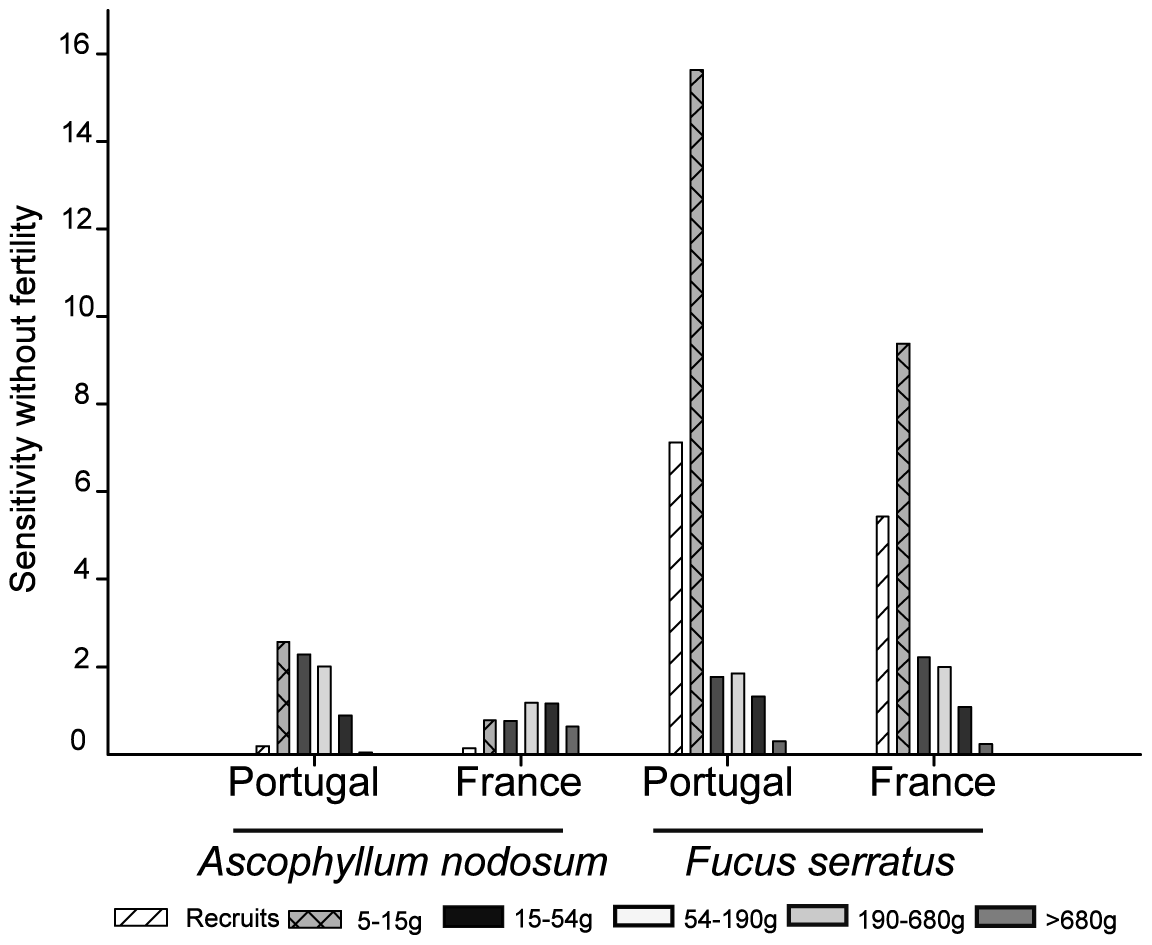
Sensitivity of the population growth rate to changes in the survival transitions for each stage class in southern (Portugal) and central (France) *A. nodosum* and *F. serratus* populations.

The pattern of sensitivities of the stochastic population growth rate to changes in the matrix elements showed that, globally, there was a selection for individual growth in both species at both locations. However, for *A. nodosum* the sensitivity to changes in loop and shrinkage transitions were also important for population growth, while the sensitivity to changes in growth transition was totally dominant over the other matrix elements in *F. serratus* (Fig. 7). In general, the sensitivity of the stochastic population growth rate to changes in fertility was very low, in particular for *F. serratus*. For *A. nodosum*, fertility transition has higher sensitivity in Portugal than in France (Fig. 7). For *A. nodosum* the sensitivity to changes in growth and loop transitions were higher in Portugal while the shrinkage transition was slightly more important in France. The comparison between the sensitivities, without taking into account the fertility across size classes, showed that the sensitivity of the stochastic population growth rate was higher for changes in classes 2 to 4 for *A. nodosum* in Portugal, while in France it was in general lower and balanced between classes 2 and 6 (Fig. 8). For *F. serratus* sensitivity of λ*_s_* was much higher for changes in smaller classes (1 and 2) and higher in Portugal than in France (Fig. 8).

## Discussion

This study revealed distinct long-term demographic trends for central and edge populations of *A. nodosum* and *F. serratus*, demonstrating the role of species identity and related life-history traits on the dynamics of edge populations. These results have wide implications for the understanding and predicting of biodiversity changes at range edges, by revealing that marginal versus central populations can have important demographic differences that determine their capacity for persistence or likelihood to shift their distributional limits.

*A. nodosum* and *F. serratus* are contrasting species in terms of life histories, habitats and genetic background. Earlier studies show that phenotypic traits are differentiated between *A. nodosum*’s southern-edge and central populations while *F. serratus* has similar traits at both locations but occupies a narrower vertical distribution in the south [12], [21]. The results of the present study show how these differences in fitness related traits are translated into the demography of the species and how sensitive the stochastic population growth rate is to changes in vital rates. An extension of this could be to use these models to predict population persistence under the future scenario of environmental variability posed by climate change.

### Stochastic Population Growth Rate and Stable Stage Distribution

For *A. nodosum*, modeled λ*_s_* was lower and more variable at the center than at the edge of distribution, but below 1 for both locations. For *F. serratus* λ*_s_* was much lower and variable at southern than at central locations. In general, macroalgae populations have lower and more variable λ*_s_* in environmentally variable habitats as a consequence of high mortality of individuals [26], [31]. An increase in the mortality of *A. nodosum* individuals has been related to different sources of disturbance such as ice scouring, harvesting and trampling [26], [32], [33]. The lower λ*_s_* found for the *A. nodosum* population in France was mainly the result of the lower survival of individuals in all size classes when compared to Portugal (see Table 1). During this study, deposition of sand was frequently observed in some of the study plots in France but never in Portugal. This episodic disturbance might have accounted for the lower survival rates observed in France. A previous study in the same area of France in the late 1990′s found much lower values of mortality and never reported accumulation of sand in the study plots, but still found λ*_s_* of less than 1 due to low recruitment rates (Åberg *et al.* personal observation). Values of λ*_s_* below 1 were reported for *A. nodosum* in the northern part of the Swedish west coast, close to its distributional limit further south on the west coast [13]. For central populations, λ*_s_* varied from slightly below to slightly above 1, depending on the location and sampling dates [13], (Åberg et al. personal observation). This demonstrates the need to conduct long-term observational studies covering a comprehensive spatial extent, along the distributional range of species, when constructing long-term predictions about population’s trends.

Southern range *F. serratus*’*s* populations show reduced plasticity to environmental variation [21]. In the present study, model simulations showed, for *F. serratus*, that environmental fluctuation at the boundary of its distribution, might cause its disappearance from previously colonized areas, which take a long time to recover [34]. The λ*_s_* values found in this study might reflect the maladaptation of populations to years with high environmental variation.

For both species, large individuals were much more abundant in France than in Portugal. The individual growth rate of both species is similar at both locations (Araújo et al. personal observation), explaining that the lower transition probability to larger size classes in Portugal is caused by higher breakage of fronds. In previous studies, increased breakage of macroalgae’s fronds has been related with several factors such as grazing [35], [36], [37], [38], wave action [39], [40], harvesting [41] and trampling [33]. However, the comparative quantitative effects of most of these factors on breakage of the fronds at central and southern locations are unknown for these species.

### Reproductive Values

The reproductive values increased with individual size for both species but more pronouncedly for *F. serratus*, especially in Portugal. This indicates that *F. serratus* individuals in Portugal have a higher fitness advantage in terms of reproduction and survival if they grow to larger size classes. An increase in the reproductive biomass with size of the plant is a common trend for seaweeds [25], [43] and an increase in the reproductive value with size class has been reported in previous studies [24], [31], [41]. Reproductive effort might increase following disturbance [44], [45], [46]. The high reproductive values of *F. serratus* in this study might reflect the levels of environmental disturbance found at its edge of distribution.

### Elasticity

Elasticity of the population growth rate to changes in survival was higher than for changes in fertility, for both species at both locations. *A. nodosum* was more susceptible to changes in loop transition followed by growth in both areas, while changes in growth transitions were the most important for *F. serratus* followed by loop. However, *F. serratus* populations were more susceptible to changes in fertility rates than those of *A. nodosum*. In general for algae, higher elasticity values for survival than for fertility are reported for longer-lived species, while in shorter-lived species elasticity values for fertility, although lower than for survival, are generally higher [13], [26], [32], [48], [47].

The higher susceptibility to changes in loop transitions of *A. nodosum* populations demonstrates that their long-term persistence might be affected by factors increasing the shrinkage of individuals (in particular of adults). Among these factors, an increase in the human frequentation of coastal areas might be important since human trampling negatively affects the communities at the southern-limit of *A. nodosum,* both in the short and long term [33]. The density of invertebrates inhabiting *A. nodosum* adults is lower in Portugal than in central and northern populations [12], [36]. However, in spite of the decreasing trend in limpet grazing from north to south [42], the opposite trend was recorded under the *A. nodosum* canopy when comparing edge and central populations [12]. Harvesting, although not important in Portugal, occurs in France, and increases individual shrinkage [32], [49]. Also changes in the frequency of storms predicted by models of climate change [50] or in the pressure of grazing [51] might account for increased shrinkage of individuals. Although the *A. nodosum* populations in Portugal were slightly more susceptible to changes in fertility than in France, elasticities for fertility transitions were low. The reproductive output [12] and reproductive allocation (Araújo et al. personal observation) of *A. nodosum* was higher in Portugal than in France, despite the apparent low importance for population growth and persistence demonstrated by the model. However, in long-lived algae, recruitment is important for the long-term stability of populations [41], [52], and massive recruitment may occur under exceptional circumstances such as disturbance events [31], [53]. Short-term studies, like the one presented here, are likely to miss these extraordinary recruitment events, potentially contributing to the occurrence of estimates of population growth below 1 observed in this study.

Factors affecting growth of individuals also have negative effects on the growth of the population, as demonstrated by the high elasticities of growth transition found for both species. The documented North Atlantic trend of raising sea and air temperatures [54] is expected to have more immediate and severe consequences at species borders. As expected, temperatures in the intertidal area are higher in Portugal than in France [12]. Increased sea surface temperature will decrease *A. nodosum* growth at temperatures above 19°C with lethal effects shown above 28°C [55]. *F. serratus* southern edge populations show much lower resilience to heat stress than central ones [21]. Consequently, it can be expected that, under the current scenario of global warming, these southern limit populations will suffer a decrease in individual growth that could have important consequences for population persistence.

### Sensitivity

The sensitivity of the stochastic population growth rate to changes in a given vital rate can be interpreted as the selection pressure on that life-history transition [22]. For both species and for both locations, the high sensitivity values of the population growth rate to changes in transition to larger size classes indicate that there is strong selection for individual growth. This selective pressure on individual growth is very pronounced for *F. serratus*, dominating over all the other transitions. For short-lived species, increased growth, directly related for *F. serratus* with its high reproductive values in large size classes, might maximize the individual life time fertility. In a scenario of increased environmental and anthropogenic disturbance, with negative effects on individual plant growth, the selective pressure for increased individual growth will be stronger. When under stressful conditions, the most beneficial trait for population persistence, in this case individual growth, will be favoured at the expenses of other fitness related traits like reproduction and defence [56], [57]. However, a reduction in the allocation of resources to defence will increase individual growth rates [58] whilst also increasing the risk of both breakage and grazing, and thus decreased net growth [58], [59]. The selective pressure on fertility is higher in Portugal than in France, although much lower than for survival transitions. Nevertheless, if the allocation of resources for growth increases, the reproductive output might be reduced with consequences for the long-term population persistence.

The results of this study show that *A. nodosum* has high plasticity in life-history traits, while *F. serratus*, with much lower and more variable λ*_s_*, seems to be more susceptible to environmental variation in local habitat conditions at the southern limit boundary. For both species, changes affecting individual growth (by diminishing the transition to larger size classes or increasing shrinkage) would reduce population growth. Under the current scenario of global environmental change, anthropogenic and environmental disturbances affecting vital rates may condition the local persistence of populations in the long term.

Future research should include studies with a particular focus on the southern biogeographic range limit of species distribution, addressing the comparison of phenotypic fitness related traits in geographically isolated versus connected edge populations.
